# ASO Author Reflections: Redefining Outcomes in Post-Mastectomy Flat Closure

**DOI:** 10.1245/s10434-025-17409-3

**Published:** 2025-05-09

**Authors:** Daniel Soroudi, Nisha Parmeshwar, Aileen Gozali, Merisa Piper

**Affiliations:** 1 School of Medicine, University of California San Francisco, San Francisco, CA USA; 2 Division of Plastic and Reconstructive Surgery, Department of Surgery, University of California San Francisco, San Francisco, CA USA

## Past

While autologous or implant-based reconstruction has long been standard following mastectomy, an increasing number of patients are opting for aesthetic flat closure.^1^ However, this population remains underrepresented in surgical outcomes research, and existing patient-reported outcome measures (PROMs), such as the BREAST-Q, were not originally designed with flat closure in mind.^2^ As a result, the quality of data available to guide shared decision-making for flat closure patients remains limited.

## Present

In the source study,^3^ a mixed-methods analysis of 252 patients undergoing post-mastectomy flat closure found a 17.5% complication rate—lower than those typically reported in implant-based and autologous reconstruction cohorts.^4^ BREAST-Q survey responses indicated high satisfaction with surgeons and physical well-being, though lower satisfaction with breasts and sexual well-being. Critically, qualitative feedback from patient emails revealed that many respondents found the BREAST-Q poorly suited to their experience. Comments highlighted concerns about triggering language, noninclusive terminology, and the irrelevance of certain modules—issues that may contribute to survey nonresponse and patient disengagement.

## Future

Improving patient-centered care includes expanding conversations around all surgical options, including flat closure. In parallel, existing PROMs such as the BREAST-Q may benefit from revision or expansion to better capture the goals and experiences of patients choosing flat closure. Our team is currently conducting a follow-up qualitative study using semi-structured interviews to further explore the limitations of the BREAST-Q from the patient perspective. Preliminary insights suggest opportunities to improve language, expand domain relevance, and enhance inclusivity. As the surgical field continues to embrace a broader range of patient priorities, redefining how we measure satisfaction is essential.
